# Quantum Representations and Scaling Up Algorithms of Adaptive Sampled-Data in Log-Polar Coordinates

**DOI:** 10.3390/e23111462

**Published:** 2021-11-04

**Authors:** Chan Li, Dayong Lu, Hao Dong

**Affiliations:** School of Mathematics and Statistics, Henan University, Kaifeng 475001, China; clchanli@163.com (C.L.); haodong96@126.com (H.D.)

**Keywords:** quantum computation, quantum data representations, data scaling up, biarcuate interpolation, log-polar coordinates

## Abstract

In log-polar coordinates, the conventional data sampling method is to sample uniformly in the log-polar radius and polar angle directions, which makes the sample at the fovea of the data denser than that of the peripheral. The central oversampling phenomenon of the conventional sampling method gives no more efficient information and results in computational waste. Fortunately, the adaptive sampling method is a powerful tool to solve this problem in practice, so the paper introduces it to quantum data processing. In the paper, the quantum representation model of adaptive sampled data is proposed first, in which the upper limit of the sampling number of the polar angles is related to the log-polar radius. Owing to this characteristic, its preparation process has become relatively complicated. Then, in order to demonstrate the practicality of the model given in the paper, the scaling up algorithm with an integer scaling ratio based on biarcuate interpolation and its circuit implementation of quantum adaptive sampled data is given. However, due to the special properties of the adaptive sampling method in log-polar coordinates, the interpolation process of adaptive sampled data becomes quite complicated as well. At the end of this paper, the feasibility of the algorithm is verified by a numerical example.

## 1. Introduction

Quantum computation [1], as the interdisciplinary subject of many disciplines, such as computer science, mathematics, physics and engineering technology, has generated much interest in the world. Quantum computation can solve many problems that classical computers cannot solve or are inefficient at, and has the ability for data processing beyond classical computers by utilizing the coherent superposition characteristics of quantum states. Quantum algorithms [2,3] use quantum interference, quantum entanglement and other unique quantum mechanical principles to calculate, which can achieve exponential acceleration or quadratic acceleration for some classical algorithms.

Quantum data processing is a pioneer in the implementation of information processing on quantum computers and has currently become a hot research field in quantum computation. Research results include: quantum data representation [4,5,6,7,8,9,10], geometric transformation [11,12], color transformation [13], feature extraction [14], data scaling [15,16,17], data watermarking [18,19], data registration [20] and so on. These research achievements have greatly promoted the development of quantum data processing.

Although quantum data processing has made great progress, there are still many problems. Most of the existing quantum data representation models are carried out in Cartesian coordinates; only few studies on quantum data representation models are discussed in log-polar coordinates. Compared with Cartesian coordinates, log-polar coordinates have the advantages of rotation and scale invariance. Because any scale and rotation in Cartesian coordinates can be represented as shifting in the log-polar radius and the polar angular directions in log-polar coordinates, respectively, it is easier to rotate data in log-polar coordinates than that in Cartesian coordinates. The existing quantum data representation model in log-polar coordinates is based on sample uniformly in both the log-polar radius and polar angle directions. The main problem is the high computational cost in data processing, which comes from central oversampling. In other words, the sample density in the data center is higher than that in the periphery, and no more useful information is gained from oversampling. An effective way to solve this problem is to employ the adaptive sampling method [21], and the detailed information is given in the next section.

According to the characteristics of log-polar coordinates, the adaptive sampling method can effectively solve the problem of oversampling in the center because the upper limit of the sampling number of the polar angles is related to the log-polar radius. This greatly avoids the computational waste caused by central oversampling. Hence, the new quantum data representation model based on the adaptive sampling method in log-polar coordinates is proposed in the paper. In quantum representations of adaptive sampled data in log-polar coordinates (QRASD), three entangled qubit sequences are applied to deposit the data information, the log-polar radius information and the polar angle information. In order to demonstrate the practicality of the representation model, the quantum data scaling up algorithm with integer scaling ratio based on biarcuate interpolation is given. The feasibility of the algorithm is verified by an example at the end of the paper.

The rest of this paper is arranged as follows. In Section 2, the conventional sampling method and adaptive sampling method in log-polar coordinates are introduced. The novel representation model and its preparation procedure are described in Section 3. In Section 4, the quantum data scaling up algorithm with integer scaling ratio using biarcuate interpolation based on the QRASD model is given. Finally, a conclusion is presented in Section 5.

## 2. Preliminaries

In this section, the conventional sampling method and adaptive sampling method in log-polar coordinates are introduced in detail first, and then the QUALPI model based on the conventional sampling method is also discussed.

**Figure 1:**
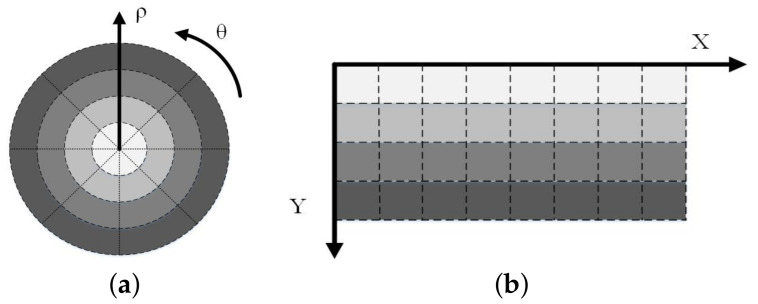
(**a**) Sampling in log-polar coordinates. (**b**) Sampling in Cartesian coordinates.

The conventional mathematical expression for a mapping procedure in which log-polar coordinates are interchangeable with Cartesian coordinates is shown below:

(1) $$\rho =\log_{base}\sqrt{{\left(x-x_{c}\right)}^{2}+{\left(y-y_{c}\right)}^{2}},$$

(2) $$\theta =\tan^{-1}\frac{y-y_{c}}{x-x_{c}},$$

where $\left(x_{c},y_{c}\right)$ represents the center datum of the transformation in Cartesian coordinates; (x,y) denotes the sampling datum in Cartesian coordinates; (ρ,θ) denotes the log-polar radius and the polar angular position in log-polar coordinates. For the sake of simplicity, the base is the natural logarithm. Figure 1 shows a conventional comparison between the two sampling methods. Figure 1a represents the conventional sampling method in log-polar coordinates, and Figure 1b represents the conventional sampling method in Cartesian coordinates.

Figure 1a indicates that the sample at the fovea of the data is denser than that of the peripheral using the conventional sampling method in log-polar coordinates, so the data processing focuses more on the fovea or central area while the peripheral area is given less consideration. Fortunately, the adaptive sampling method in log-polar coordinates [21] avoids this problem, which is introduced below.

Let $n_{\rho }$ and $n_{\theta }$ be the numbers of samples in the log-polar radius and the polar angular directions, respectively; $R_{i}$, $i=0,1,\ldots ,n_{\rho }$, be the radius size which is sampled equally to cover $360^{\circ }$ in the angular direction for $\theta =0,1,\ldots ,n_{\theta }$. Furthermore, to prevent undersampling in the log-polar radius direction, $R_{n_{\rho }}-R_{n_{\rho }-1}\leq 1$ must be satisfied. It is easy to see that the following equation should be true:

(3) $$R_{i}=e^{\frac{i\times \log R_{max}}{n_{\rho }}},\;R_{max}=R_{n_{\rho }},$$

(4) $$n_{\rho }\geq \frac{\log R_{max}}{\log R_{max}-\log\left(R_{max}-1\right)}.$$

In the polar angular direction, the maximum sampling circumference, $R_{max}$, has approximately $2\pi R_{max}$ data. Hence, the number of samples in the polar angular direction is equal or greater than that amount, which is necessary to prevent data being missed in the sampling procedure, that is:

(5) $$n_{\theta }\geq 2\pi R_{max}.$$

**Figure 2:**
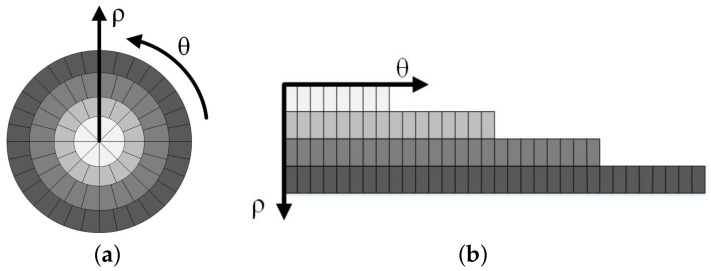
(**a**) Adaptive sampling in log-polar coordinates. (**b**) The resulting sample distribution in the polar angular and log-polar radius directions.

To make the computation simpler, $n_{\rho }$ and $n_{\theta }$ are selected as a multiple power of two. Then, Equations (4) and (5) can be expressed as:

(6) $$n_{\rho }=2^{\left\lceil \frac{1}{\log2}\times \log\frac{\log R_{max}}{\log R_{max}-\log\left(R_{max}-1\right)}\right\rceil },\;n_{\theta }=8R_{max},$$

where ⌈A⌉ is the nearset integer equal to or greater than A. It is found that the most suitable sampling method is to set the number of polar angles to eight times the number of log-polar radii. Therefore, the adaptive sampling method uses an eight-fold sampling method for each circumference. The adaptive sampling method is shown in Figure 2a. Figure 2b describes the resulting sample distribution in the polar angular and log-polar radius directions, a series of sample points are arranged in the step-like manner, that is, as ρ increases, the length of the sample points (number of samples represented by θ) increases. The total number of samples is less than what is needed for the conventional sampling method to have the same sampling resolution.

The QUALPI model [9] based on conventional sampling method stores and processes data in log-polar coordinates. It utilizes three entangled qubit sequences to store the gray scale information, the log-polar radius position and the polar angular position information for each pixel in a log-polar image. The model is shown below:

(7) $$\begin{array}{c} |I\rangle =\frac{1}{{\sqrt{2}}^{m+n}}\sum_{\rho =0}^{2^{m}-1}\sum_{\theta =0}^{2^{n}-1}|g\left(\rho ,\theta \right)\rangle ⊗|\rho \rangle ⊗|\theta \rangle , \end{array}$$

(8) $$g\left(\rho ,\theta \right)=C_{\rho \theta }^{0}C_{\rho \theta }^{1}\ldots C_{\rho \theta }^{q-1},\;C_{YX}^{t}\in \left\{0,1\right\},\;g\left(\rho ,\theta \right)\in \left[0,2^{q}-1\right].$$

The QUALPI model realizes the quantum representation of log-polar images for the first time, but the problem of central oversampling in log-polar coordinates still exists. Therefore, it is of great significance for the development of quantum data processing in the future to improve the existing quantum data representation models and find a better method to represent quantum data in log-polar coordinates.

## 3. Quantum Data Representations and Preparation Based on Adaptive Sampling Method

The premise of the application of data in log-polar coordinates is the existence of its representation and preparation. In this section, details of representations and preparation procedures are given in Section 3.1 and Section 3.2, respectively. The time complexity of the preparation procedure is also discussed in Section 3.3.

### 3.1. Quantum Data Representations Based on Adaptive Sampling Method

Motivated by the QUALPI model, a novel quantum representation model of adaptive sampled-data in log-polar coordinates (QRASD) to store and process data is proposed as follows:

(9) $$|D\rangle =\frac{1}{{\sqrt{2}}^{2m+3}}\sum_{\rho =0}^{M-1}\sum_{\theta =0}^{8\rho +7}|f\left(\rho ,\theta \right)\rangle |\rho \rangle |\theta \rangle ,$$

(10) $$m=\left\{\begin{array}{cc} \left\lceil \log_{2}M\right\rceil , & M>1;\\ 1, & M=1, \end{array}\right.$$

where ρ encodes the information of log-polar radius and θ encodes the information of the polar angle. For each fixed ρ, i.e., in the circumference with a log-polar radius ρ, the circumference is sampled equidistantly with sampling number of 8ρ and sampling interval of 2π/8(ρ+1). It is worth noting that both ρ and θ are coded from 0, which have a certain impact on subsequent calculations.

In order to improve the flexibility and breadth of the model, three different data information representation methods are presented.

1. *Representing the data information as in the FRQI model*

When we replace |f(ρ,θ)〉 in Equation (9) with the method given in the FRQI model [7], i.e., $|f\left(\rho ,\theta \right)\rangle =\cos\alpha_{\rho \theta }|0\rangle +\sin\alpha_{\rho \theta }|1\rangle$, then we have:

(11) $$|D\rangle =\frac{1}{{\sqrt{2}}^{2m+3}}\sum_{\rho =0}^{M-1}\sum_{\theta =0}^{8\rho +7}(\cos\alpha_{\rho \theta }|0\rangle +\sin\alpha_{\rho \theta }|1\rangle )|\rho \rangle |\theta \rangle ,$$

(12) $$\alpha_{\rho \theta }\in \left[0,\frac{\pi }{2}\right],\;\rho =0,1,\ldots ,M-1,\;\theta =0,1,\ldots ,8\rho +7.$$

Equation (11) is referred to as the FRQI model based on the adaptive sampling method, shorthand for the AS-FRQI sub-model.

2. *Representing the data information as in the NEQR model*

If |f(ρ,θ)〉 is taken the form of the data information in the NEQR model [8], where |f(ρ,θ)〉 = $|C_{\rho \theta }^{0}C_{\rho \theta }^{1}\ldots C_{\rho \theta }^{q-1}\rangle$, $C_{\rho \theta }^{t}\in \left\{0,1\right\}$, t=0,1,…,q−1, then Equation (9) can be rewritten as:

(13) $$|D\rangle =\frac{1}{{\sqrt{2}}^{2m+3}}\sum_{\rho =0}^{M-1}\sum_{\theta =0}^{8\rho +7}\overset{q-1}{\underset{t=0}{⨂}}|C_{\rho \theta }^{t}\rangle |\rho \rangle |\theta \rangle .$$

The new representation method is called the NEQR model based on an adaptive sampling method, or the AS-NEQR sub-model for short.

3. *Representing the data information as in the QR2-DD model*

**Figure 3:**
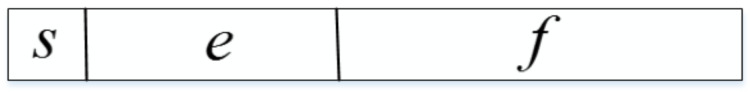
Format of floating-point numbers in IEEE-754.

The IEEE (Institute of Electrical and Electronics Engineers) is an international organization that has designed specific binary formats for storing floating-point numbers. IEEE-754 is the most widely used standard for floating point arithmetic operations. Figure 3 shows the format of floating-point numbers in IEEE-754.

The QR2-DD model [10] uses the IEEE-754 standard to represent quantum data information, which lays a foundation for quantum data processing. Let |f(ρ,θ)〉= $|s_{\rho \theta }^{0}s_{\rho \theta }^{1}\ldots s_{\rho \theta }^{p}s_{\rho \theta }^{p+1}\ldots s_{\rho \theta }^{p+q-1}\rangle$, $s_{\rho \theta }^{t}\in \left\{0,1\right\}$, then Equation (9) goes to the following form:

(14) $$|D\rangle =\frac{1}{{\sqrt{2}}^{2m+3}}\sum_{\rho =0}^{M-1}\sum_{\theta =0}^{8\rho +7}\overset{p+q-1}{\underset{t=0}{⨂}}|s_{\rho \theta }^{t}\rangle |\rho \rangle |\theta \rangle ,$$

where $S_{YX}^{s}=s_{YX}^{0}$ is the sign qubit, $S_{YX}^{e}=s_{YX}^{1}s_{YX}^{2}\ldots s_{YX}^{p}$ encodes the exponent and $S_{YX}^{f}=s_{YX}^{p+1}s_{YX}^{p+2}\ldots s_{YX}^{p+q-1}$ encodes the mantissa. The QR2-DD model based on adaptive sampling method is abbreviated as the AS-QR2-DD sub-model.

**Figure 4:**
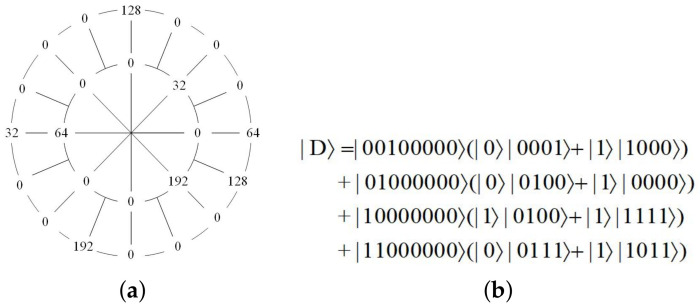
(**a**) A 2×16 adaptive sampled-data in log-polar coordinates. (**b**) Its quantum representation expression according to the AS-NEQR sub-model.

Figure 4a takes a ρ×8ρ(ρ=0,1) quantum data |D〉 as an example, that is, when ρ=0, there is θ=0,1,…,7, and when ρ=1, there is θ=0,1,…,15. Its quantum representation expression based on the AS-NEQR sub-model is given in Figure 4b. The data information of |D〉 from big to small are 192, 128, 64, 32 and 0. The quantum representation expression omits the part where the data is 0.

### 3.2. Quantum Data Preparation Based on Adaptive Sampling Method

Since the preparation process for the three quantum data representation sub-models given above is similar, only one detailed preparation process is given in this subsection. Compared with the AS-FRQI sub-model and the AS-NEQR sub-model, floating-point numbers can represent decimal numbers, which is more suitable for data processing in actuality. Therefore, the AS-QR2-DD sub-model is taken as an example for quantum preparation.

**Figure 5:**
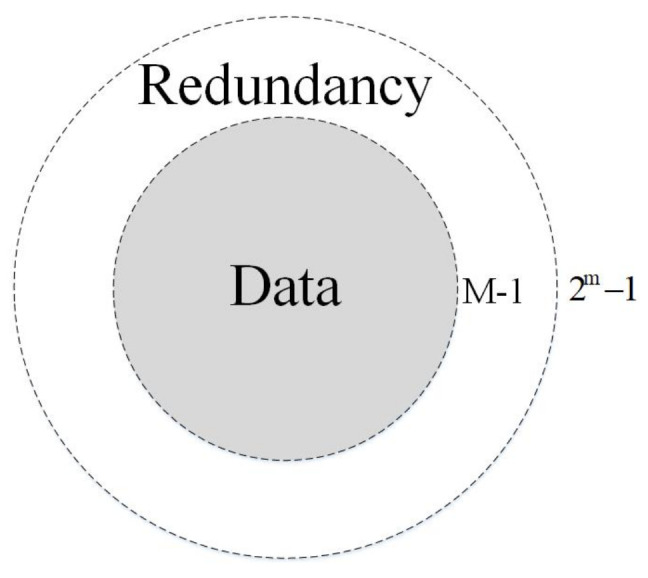
The data information and the redundancy.

Due to the inherent characteristics of the binary operation process of quantum computer, redundancy is an inevitable result in the process of binary computation in quantum computers. In Figure 5, the gray part contains data information, while the white part is redundant without data information.

The empty quantum state with (p+q+2m+3) qubits is constructed as follows:

(15) $$|\psi_{0}{\rangle =|0\rangle }^{⨂(p+q+2m+3)}.$$

**Figure 6:**
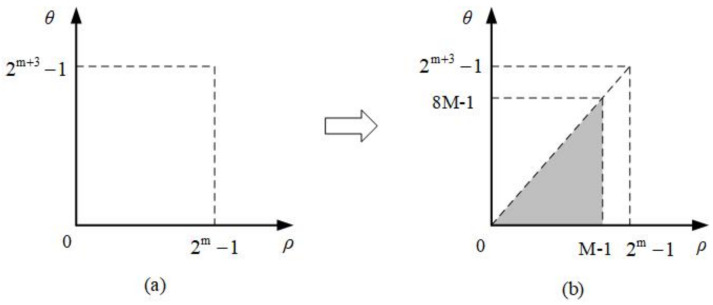
(**a**) Prepare position information. (**b**) Prepare data information.

The preparation process of adaptive sampled-data is shown in Figure 6. With the empty quantum state, the position information is first prepared in Figure 6a, and then the data information, which is the shaded part in Figure 6b, is prepared by using the comparator.

Step 1: In this step, the single-qubit identity gate *I* and the Hadamard gate *H* are utilized to transform $|\psi_{0}\rangle$ to the intermediate state $|\psi_{1}\rangle$. The whole quantum operation $U_{1}$ in Step 1 is defined as follows:

(16) $$U_{1}=I^{⊗(p+q)}⊗H^{⊗m}⊗H^{⊗(m+3)},$$

which transforms the initial state $|\psi_{0}\rangle$ to the intermediate state $|\psi_{1}\rangle$:

(17) $$\begin{array}{c} |\psi_{1}\rangle =U_{1}(|\psi_{0}\rangle )=\frac{1}{{\sqrt{2}}^{2m+3}}\sum_{\rho =0}^{2^{m}-1}\sum_{\theta =0}^{2^{m+3}-1}{|0\rangle }^{⊗(p+q)}|\rho \rangle |\theta \rangle . \end{array}$$

Step 2: Compared with the preparation process of the existing quantum data representation models, the upper limit of the sampling number of θ in the QRASD model is related to ρ, that is 0≤θ≤(8ρ+7), so a comparison between (8ρ+7) and θ is necessary. In order to achieve this goal, some quantum circuit modules are introduced first.

1. *Plus one and minus one modules*

**Figure 7:**
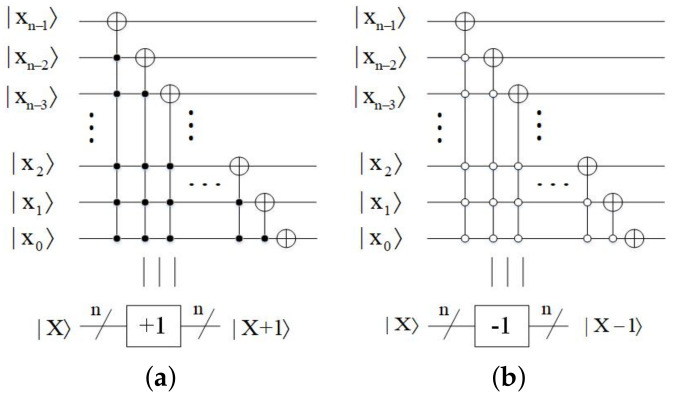
(**a**) Quantum circuit of plus one module. (**b**) Quantum circuit of minus one module.

This part is designed to perform plus one and minus one [17] operations. The quantum circuit of plus one and minus one modules are shown in Figure 7a,b, respectively.

2. *Multiply by eight module*

**Figure 8:**
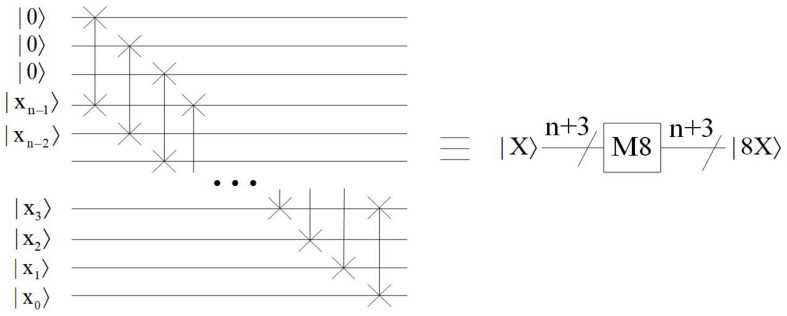
Quantum circuit for multiplying by eight module.

This module implements the multiplication by eight, i.e., |X〉→|8X〉, and the quantum circuit is shown in Figure 8. The essence of multiplying by eight is that the qubit shifts three places to the left. It can be seen that, the effect of the module is $|000x_{n-1}x_{n-2}\ldots x_{0}\rangle$→$|x_{n-1}x_{n-2}\ldots x_{0}000\rangle$.

3. *Comparator module*

**Figure 9:**
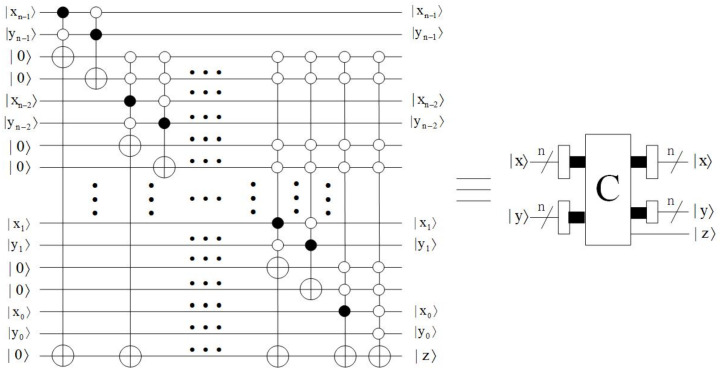
Quantum circuit of comparator module.

Comparator [22] plays an important role in quantum data processing. The function of this module is to compare |x〉 and |y〉, where |x〉 = $|x_{n-1}x_{n-2}\ldots x_{0}\rangle$, |y〉 = $|y_{n-1}y_{n-2}\ldots y_{0}\rangle$, $x_{t}$, $y_{t}\in \left\{0,1\right\}$, *t* = 0,1,…,n−1. The qubit |z〉 is the output. If |z〉=|1〉, then x≥y; if |z〉=|0〉, then x<y. The quantum circuit of the comparator module is shown in Figure 9.

**Figure 10:**
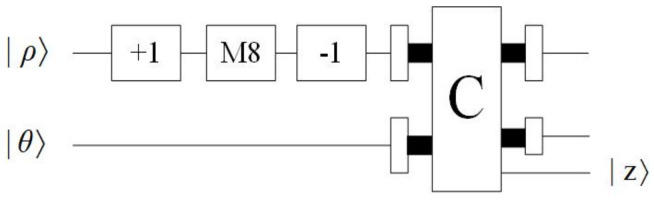
Quantum circuit of Step 2.

These are all the modules required for the process of comparison. As shown in Figure 10, the specific operation of this step is as follows: The qubit representing ρ is executed first by the operation of adding one to obtain (ρ+1). Then, (ρ+1) is multiplied by eight and subtracted by one to obtain (8ρ+7). Finally, (8ρ+7) is compared with θ. When the output |z〉 is equal to |1〉, the next step is executed.

Step 3: Since there are 8(ρ+1) polar angles on each circumference of log-polar radius ρ, this step is divided into 8(1+2+…+M)=4M(M+1) sub-operations to set the data information at the corresponding position. The sub-operation $U_{\rho \theta }$ is expressed as below:

(18) $$U_{\rho \theta }=I^{⊗(p+q)}⊗\underset{\left(l,k)\neq (\rho ,\theta \right)}{\sum_{l=0}^{2^{m}-1}\sum_{k=0}^{2^{m+3}-1}}|lk\rangle \langle lk|+\Omega_{\rho \theta }⊗|\rho \theta \rangle \langle \rho \theta |,$$

(19) ρ=0,1,…,M−1,θ=0,1,…,8ρ+7,

where the value-setting operation $\Omega_{\rho \theta }$ can be shown as:

(20) $$\Omega_{\rho \theta }=\overset{p+q-1}{\underset{t=0}{⨂}}\Omega_{\rho \theta }^{t},\;\Omega_{\rho \theta }^{t}:|0\rangle \to |0⊕s_{\rho \theta }^{t}\rangle .$$

It is obviously that $\Omega_{\rho \theta }$ only affect data at the specified position. The process for $\Omega_{\rho \theta }$ to obtain data is as follows:

(21) $$\Omega_{\rho \theta }{|0\rangle }^{⊗(p+q)}=\overset{p+q-1}{\underset{t=0}{⨂}}\left(\Omega_{\rho \theta }^{t}|0\rangle \right)=\overset{p+q-1}{\underset{t=0}{⨂}}|0⊕s_{\rho \theta }^{t}\rangle =\overset{p+q-1}{\underset{t=0}{⨂}}|s_{\rho \theta }^{t}\rangle =|f\left(\rho ,\theta \right)\rangle .$$

The following formula shows the result of the intermediate state $|\psi_{1}\rangle$ under the influence of $U_{\rho \theta }$.

(22) $$\begin{array}{ccc} U_{\rho \theta }(|\psi_{1}\rangle ) & = & \frac{1}{{\sqrt{2}}^{2m+3}}\left(\underset{\left(l,k)\neq (\rho ,\theta \right)}{\sum_{l=0}^{2^{m}-1}\sum_{k=0}^{2^{m+3}-1}}{|0\rangle }^{⊗(p+q)}|l\rangle |k\rangle +\Omega_{\rho \theta }{|0\rangle }^{⊗(p+q)}|\rho \rangle |\theta \rangle \right)\\ & = & \frac{1}{{\sqrt{2}}^{2m+3}}\left(\underset{\left(l,k)\neq (\rho ,\theta \right)}{\sum_{l=0}^{2^{m}-1}\sum_{k=0}^{2^{m+3}-1}}{|0\rangle }^{⊗(p+q)}|l\rangle |k\rangle +|f\left(\rho ,\theta \right)\rangle |\rho \rangle |\theta \rangle \right). \end{array}$$

To set the 4M(M+1) log-polar data, the whole operation $U_{2}$ which transforms the intermediate state $|\psi_{1}\rangle$ to the final state $|\psi_{2}\rangle$ is defined below:

(23) $$U_{2}=\prod_{\rho =0}^{M-1}\prod_{\theta =0}^{8\rho +7}U_{\rho \theta },$$

(24) $$\begin{array}{ccc} |\psi_{2}\rangle & = & U_{2}\left(|\psi_{1}\rangle \right)\\ & = & \frac{1}{{\sqrt{2}}^{2m+3}}\sum_{\rho =0}^{M-1}\sum_{\theta =0}^{8\rho +7}|f\left(\rho ,\theta \right)\rangle |\rho \rangle |\theta \rangle . \end{array}$$

Hitherto, the initialized state ${|0\rangle }^{⨂(p+q+2m+3)}$ is transformed to the desired state.

### 3.3. Time Complexity Analysis of the Preparation Procedure

Time complexity refers to the time needed to complete an algorithm in the field of computer science and engineering, and is an important parameter to evaluate the merits of an algorithm. Therefore, it is necessary to discuss the time complexity of the preparation procedure. To calculate the time complexity, the number of operation units of the preparation procedure must be estimated, and each operation unit runs for the same amount of time.

**Theorem** **1.** *In order to store ρ×8ρ (ρ=0,1,…,M−1) data with a data range from 0 to $2^{p+q}-1$, the whole quantum preparation of the AS-QR2-DD sub-model costs no more than $O(m^{2}+\left(p+q\right)mM^{2})$.*

**Proof.**  Firstly, in Step 1, $U_{1}$ is consisted of (2m+3) Hadamard gates, and:

(25) $$m=\left\{\begin{array}{cc} \left\lceil \log_{2}M\right\rceil , & M>1;\\ 1, & M=1, \end{array}\right.$$

where ⌈A⌉ is the nearset integers equal to or greater than A. Then it’s obvious that the time complexity of this step is O(m).

Secondly, $U_{2}$ can be decomposed into four operations: plus one, multiply by eight, minus one and comparator operations.

**Figure 11:**
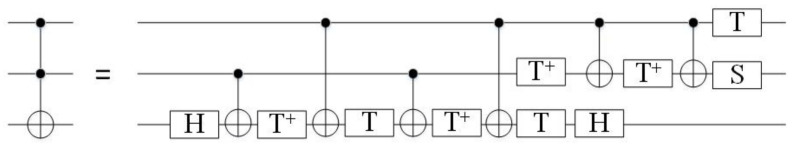
Toffoli gate is decomposed into basic quantum gates.

The plus one module consists of one NOT gate, one *C*-NOT gate, one $C^{2}$-NOT gate, …, and one $C^{m-1}$-NOT gate. While a Toffoli gate ($C^{2}$-NOT gate) can be decomposed into six *C*-NOT gates and some 1-bit gates, such as two Hadamard gates *H*, one phase gate *S*, three $\frac{\pi }{8}$ gates *T* and four conjugate transpose of the $\frac{\pi }{8}$ gate $T^{+}$ [23]. Summed up, a Toffoli gate has a time complexity of 16. Figure 11 depicts the process of implementing the Toffoli gate with elementary quantum gates. When t=3 and 4, one $C^{t}$-NOT gate can be decomposed into $\left(3\cdot 2^{t}-4\right)$ *C*-NOT gates and $2\cdot 2^{t}$ 1-bit gates. When t=5,6,…,m−1, each controlled quantum gate $C^{t}$-NOT can be simulated by 8(t−3) Toffoli gates [24]. Therefore, the time complexity of the plus one module is $O(m^{2})$.

Multiplied by the eight module contains *m* swap gates, which can be decomposed into three *C*-NOT gates. So the time complexity of multiplying by the eight module is O(m).

Similar to the plus one module, the minus one module requires one NOT gate, one *C*-NOT gate, one $C^{2}$-NOT gate, …, and one $C^{m+2}$-NOT gate to complete. Then, the time complexity of the minus one module is also $O(m^{2})$.

The comparator operation consists of one $C^{2(m+3)}$-NOT gate, one $C^{2m+5}$-NOT gate, three $C^{2}$-NOT gates, three $C^{4}$-NOT gates, …and three $C^{2(m+2)}$-NOT gates. The further decomposition has the time complexity of the comparator module $O(m^{2})$.

To sum up, the time complexity of Step 2 is $O(m^{2})$.

Last but not least, the operation $U_{2}$ in Step 3 can be divided into 4M(M+1) sub-operations $U_{\rho \theta }$ to set all the data at the corresponding position. Each $U_{\rho \theta }$ is composed of (p+q) $C^{2m+3}$-NOT gates. It is known that sub-operation $U_{\rho \theta }$ costs no more than O((p+q)m). Hence, the time complexity of Step 3 will cost no more than $O(\left(p+q\right)mM^{2})$.

As the discussion above, the time complexity of the whole preparation procedure is about $O(m^{2}+\left(p+q\right)mM^{2})$. □

**Figure 12:**
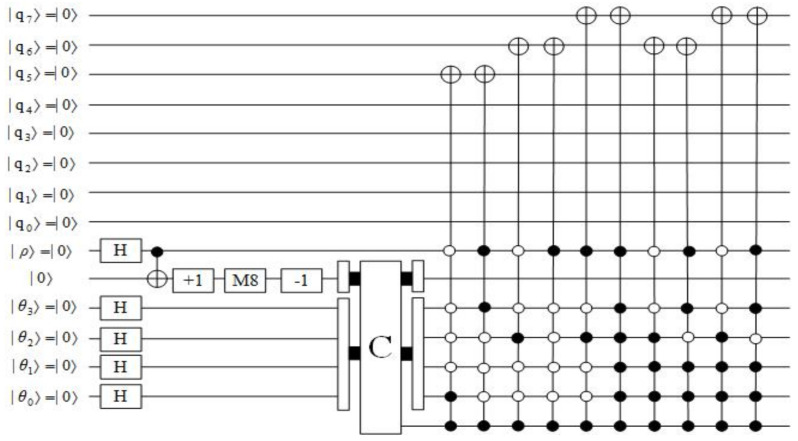
The quantum circuit of preparation for the example data shown in Figure 4.

The quantum circuit of preparation for the example data shown in Figure 4 is described in Figure 12.

## 4. Quantum Data Scaling Up Algorithm with Integer Scaling Ratio Based on Biarcuate Interpolation

Data scaling up as a kind of data geometric transformation has been widely studied and applied in the classical data processing; however, the quantum version of which, in log-polar coordinates does not exist. Here, by employing biarcuate interpolation, a quantum algorithm to scale up adaptive sampled-data in log-polar coordinates is given. To make it easier to understand, the AS-NEQR sub-model is taken as an example.

### 4.1. Quantum Circuit Modules for Arithmetic Operations

Designing a quantum scaling up circuit of biarcuate interpolation is a complex task. A series of quantum circuit modules to realize special functions are introduced first, including multiplying the *C*-NOT operation module, adder module [25], subtractor module [25], multiplier module [26] and divider module [27].

#### 4.1.1. Multiply *C*-NOT Operation Module

**Figure 13:**
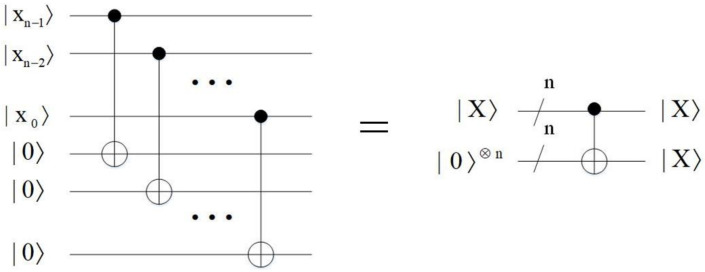
Quantum circuit of multiply *C*-NOT operation.

The multiply *C*-NOT operation is to copy the determined quantum states. The quantum circuit of the multiply *C*-NOT operation is shown in Figure 13. It utilizes *nC*-NOT gates to copy the *n*-qubit information of $\left|X\rangle =\right|x_{n-1}x_{n-2}\ldots x_{0}\rangle$ into the *n* ancillary qubits ${|0\rangle }^{⊗n}$, where $|x_{n-1}\rangle$, $|x_{n-2}\rangle$, …, $|x_{1}\rangle$ and $|x_{0}\rangle$ are the control qubits and the *n* ancillary qubits ${|0\rangle }^{⊗n}$ are the target qubits. The time complexity of the multiply *C*-NOT operation module is O(n).

#### 4.1.2. Adder Module

**Figure 14:**
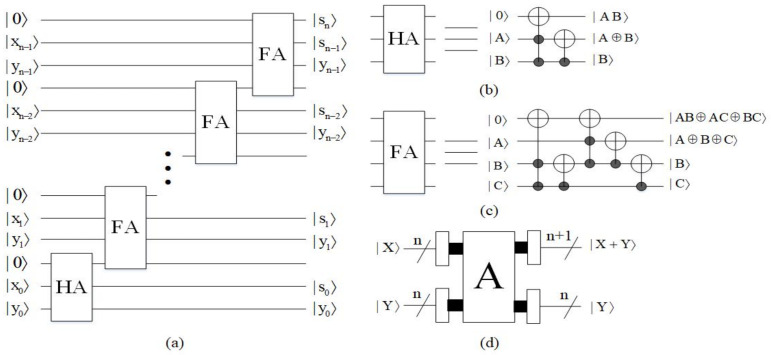
(**a**) Quantum circuit of adder. (**b**) Quantum circuit of half adder. (**c**) Quantum circuit of full adder. (**d**) Quantum simple circuit of adder.

The adder module [25] is not only the core of the multiplier, but also one of the core modules of constructing the biarcuate interpolation method. The adder (A) adding a *n*-qubit unsigned integer |Y〉 to a *n*-qubit unsigned integer |X〉 is designed by one half adder (HA) and (n−1) full adders (FA) as shown in Figure 14. Here, the input |X〉 comes out as the sequence $|s_{n}s_{n-1}\ldots s_{0}\rangle$, which represents the sum of |X+Y〉, and the input |Y〉 stays the same. Other unremarked qubits are the garbage outputs and the input qubit |0〉 is the ancillary constant input. For convenience, the block diagram of A omits the ancillary inputs and the garbage outputs. The time complexity of the adder module is O(n).

#### 4.1.3. Subtractor Module

**Figure 15:**
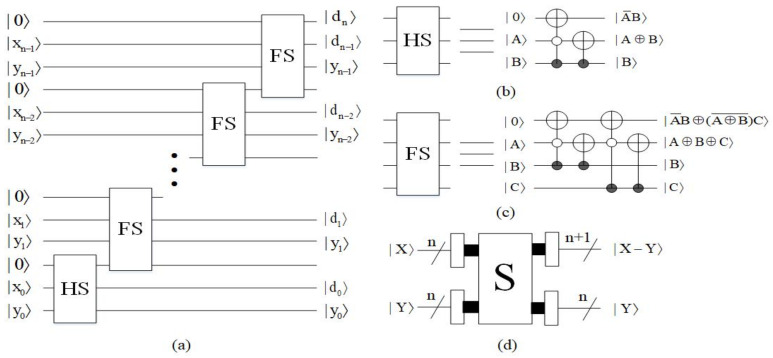
(**a**) Quantum circuit of subtracter. (**b**) Quantum circuit of half subtracter. (**c**) Quantum circuit of full subtracter. (**d**) Quantum simple circuit of subtracter.

The function of the subtractor (S) is to implement the subtraction of binary unsigned integers |X〉 and |Y〉 [25]. The subtractor is designed by one half subtractor (HS) and (n−1) full subtractors (FS). The input |X〉 come out as the sequence $|d_{n}d_{n-1}\ldots d_{0}\rangle$, which represents the difference of |X−Y〉, and the input |Y〉 stays the same. If X≥Y, then $|d_{n}\rangle =|0\rangle$, if X<Y, then $|d_{n}\rangle =|1\rangle$. The quantum circuit of the subtractor module is shown in Figure 15. The time complexity of the subtractor module is O(n).

#### 4.1.4. Multiplier Module

The purpose of the multiplier module [26] is to take the product of two numbers, which can be done by addition. Let $\left|X\rangle =\right|x_{m-1}x_{m-2}\ldots x_{0}\rangle$ and $\left|Y\rangle =\right|y_{n-1}y_{n-2}\ldots y_{0}\rangle$, then:

(26) $$\begin{array}{ccc} |X\times Y\rangle & = & |\left(2^{m-1}x_{m-1}+2^{m-2}x_{m-2}+\ldots +2^{0}x_{0}\right)\times Y\rangle \\ & = & |\left(2^{m-1}x_{m-1}\right)\times Y+\left(2^{m-2}x_{m-2}\right)\times Y+\ldots +\left(2^{0}x_{0}\right)\times Y\rangle \\ & = & |x_{m-1}\times \left(2\times 2^{m-2}Y\right)+x_{m-2}\times \left(2\times 2^{m-3}Y\right)+\ldots +x_{0}\times \left(2^{0}\times Y\right)\rangle . \end{array}$$

**Figure 16:**
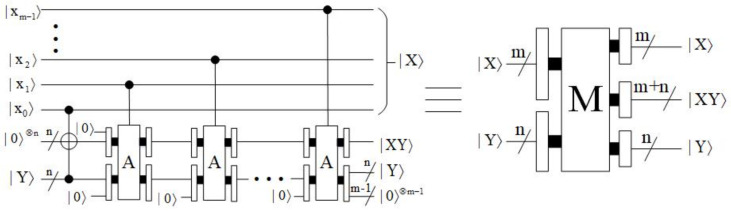
Quantum circuit of multiplier module.

First, the auxiliary qubit ${|0\rangle }^{⊗n}$ is given as the initial value of the partial product. When $|x_{0}\rangle =|1\rangle$, copy |Y〉 to the partial product, add ‘0’ to the left of the partial product, and add ‘0’ to the right of |Y〉. Then, if $|x_{t}\rangle =|1\rangle$, t=1,2,…,m−1, add the padded |Y〉 to the partial product. If $|x_{t}\rangle =|0\rangle$, the partial product remains unchanged. Since the adder is a carry addition, only after the proccess do we need to add ‘0’ to the right of |Y〉. The block diagram of multiplier (M) is shown in Figure 16, where it consists of multiple adders. The time complexity of the multiplier module is O(mn).

#### 4.1.5. Divider Module

**Figure 17:**
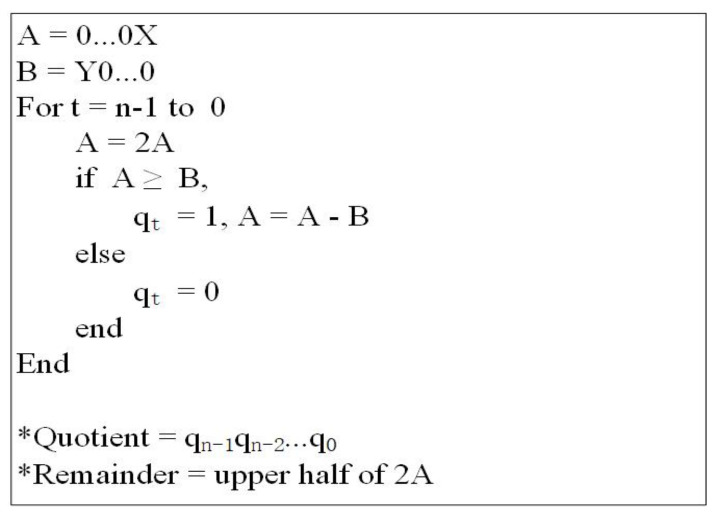
Pseudo code for the division process.

Based on restoring the division algorithm, Khosropour et al. [27] proposed the quantum division circuit. In order to make full use of the operation modules given in this paper, appropriate modifications have been made in this subsection. In Figure 17, a pseudo code for this algorithm is given.

According to the algorithm, to get the quotient (|Q〉) and the remainder (|R〉), first of all, the lowest *n*-qubit of |A〉 are equal to the dividend |X〉, and the highest *n*-qubit are equal to zero. Furthermore, register |B〉 is initiated such that its highest *n*-qubit contains the divisor |Y〉 and its lowest *n*-qubit are set to zero. Then, |A〉 is multiplied by two and compared to |B〉. If the result is nonnegative, then the qubit $|q_{t}\rangle$ in the quotient is set to one and |A〉 should be subtracted |B〉; otherwise, $|q_{t}\rangle =|0\rangle$. This process is repeated *n* times, each time one qubit of the quotient is determined. A concrete calculation example is given to clarify this procedure. Let |X〉=|1001〉 and |Y〉=|0010〉, then the initial state is:

$$\begin{array}{c} |A\rangle =|00001001\rangle ,\;|B\rangle =|00100000\rangle . \end{array}$$

The first iteration:

$$\begin{array}{c} |A\rangle =|2A\rangle =\left|00010010\rangle ,\;|A\rangle <|B\rangle ,\;\right|q_{3}\rangle =|0\rangle . \end{array}$$

The second iteration:

$$\begin{array}{c} |A\rangle =|2A\rangle =|00100100\rangle ,\;|A\rangle \geq |B\rangle . \end{array}$$

$$\begin{array}{c} |q_{2}\rangle =|1\rangle ,\;|A\rangle =|A-B\rangle =|00000100\rangle . \end{array}$$

The third iteration:

$$\begin{array}{c} |A\rangle =|2A\rangle =\left|00001000\rangle ,\;|A\rangle <|B\rangle ,\;\right|q_{1}\rangle =|0\rangle . \end{array}$$

The forth iteration:

$$\begin{array}{c} |A\rangle =|2A\rangle =\left|00010000\rangle ,\;|A\rangle <|B\rangle ,\;\right|q_{0}\rangle =|0\rangle . \end{array}$$

Therefore, it turns out that:

$$\begin{array}{c} |R\rangle =|0001\rangle ,\;|Q\rangle =|0100\rangle . \end{array}$$

**Figure 18:**
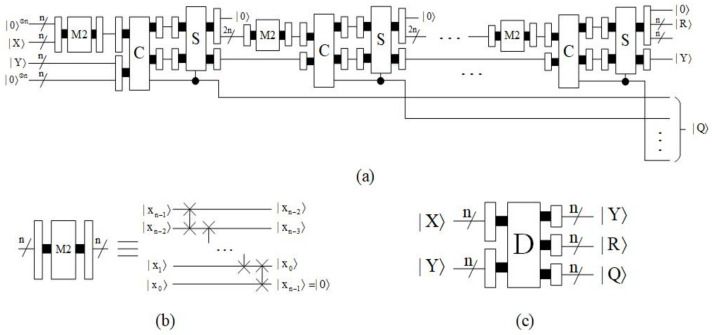
(**a**) Quantum circuit of divider. (**b**) Quantum circuit of multiply by two. (**c**) Quantum simple circuit of divider.

Obviously, the calculation process of the divider can be achieved through the comparators, subtractors and multiply by two operations. The corresponding quantum circuit is shown in Figure 18. In order to facilitate the subsequent operations, the upper output is marked as Y, the middle output is marked as R and the lower output is marked as Q in the quantum simple circuit of the divider. The time complexity of the divider module is $O(n^{3})$.

### 4.2. Quantum Biarcuate Interpolation Method in Log-Polar Coordinates

The principle of the biarcuate interpolation method in log-polar coordinates is similar to that of the bilinear interpolation method in Cartesian coordinates. The difference is that bilinear interpolation is based on line segments, but biarcuate interpolation is based on angular segments.

#### 4.2.1. Arcuate Interpolation Method

**Figure 19:**
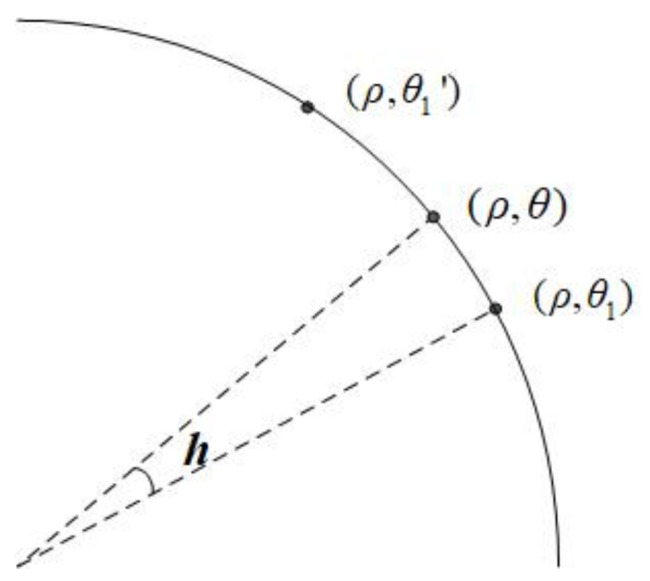
Arcuate interpolation method.

The datum at position (ρ,θ) of the interpolated data can be reconstructed from the datum at positions $\left(\rho ,\theta_{1}\right)$ and $\left(\rho ,\theta_{1}^{\prime }\right)$ of the original data. As shown in Figure 19, the value of the desired datum f(ρ,θ) can be obtained by the following equation:

(27) $$\begin{array}{c} \frac{f\left(\rho ,\theta \right)-f\left(\rho ,\theta_{1}\right)}{\theta -\theta_{1}}=\frac{f\left(\rho ,\theta_{1}^{\prime }\right)-f\left(\rho ,\theta \right)}{\theta_{1}^{\prime }-\theta }, \end{array}$$

where $f(\rho ,\theta_{1})$ and $f(\rho ,\theta_{1}^{\prime })$ are two known data. That is to say,

(28) $$\begin{array}{c} f\left(\rho ,\theta \right)=\left(1-h\right)f\left(\rho ,\theta_{1}\right)+hf\left(\rho ,\theta_{1}^{\prime }\right),\;h=\frac{\theta -\theta_{1}}{\theta_{1}^{\prime }-\theta_{1}}. \end{array}$$

#### 4.2.2. Biarcuate Interpolation Method

**Figure 20:**
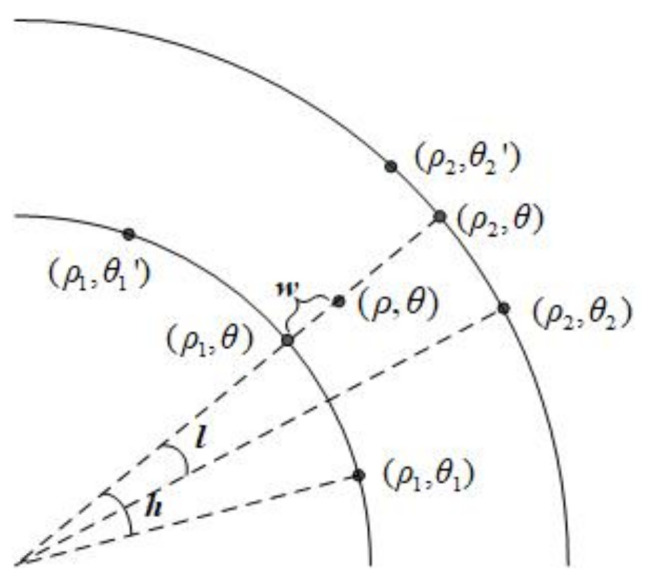
Biarcuate interpolation method.

The biarcuate interpolation method is more complicated than the arcuate interpolation method. The value f(ρ,θ) at position (ρ,θ) of the interpolated data can be reconstructed from the value at positions $\left(\rho_{1},\theta_{1}\right)$, $\left(\rho_{1},\theta_{1}^{\prime }\right)$, $\left(\rho_{2},\theta_{2}\right)$ and $\left(\rho_{2},\theta_{2}^{\prime }\right)$ of the original data. The corresponding relationship is shown in Figure 20.

To get f(ρ,θ), auxiliary points $\left(\rho_{1},\theta \right)$ and $\left(\rho_{2},\theta \right)$ are added to facilitate the calculation. The datum at position $\left(\rho_{1},\theta \right)$ can be calculated as follows:

(29) $$\frac{f\left(\rho_{1},\theta \right)-f\left(\rho_{1},\theta_{1}\right)}{\theta -\theta_{1}}=\frac{f\left(\rho_{1},\theta_{1}^{\prime }\right)-f\left(\rho_{1},\theta \right)}{\theta_{1}^{\prime }-\theta },$$

where $f(\rho_{1},\theta_{1})$ and $f(\rho_{1},\theta_{1}^{\prime })$ are given. That is to say,

(30) $$f\left(\rho_{1},\theta \right)=\left(1-h\right)f\left(\rho_{1},\theta_{1}\right)+hf\left(\rho_{1},\theta_{1}^{\prime }\right),\;h=\frac{\theta -\theta_{1}}{\theta_{1}^{\prime }-\theta_{1}}.$$

In the same manner,

(31) $$\begin{array}{cc} & f\left(\rho_{2},\theta \right)=\left(1-l\right)f\left(\rho_{2},\theta_{2}\right)+lf\left(\rho_{2},\theta_{2}^{\prime }\right),\;l=\frac{\theta -\theta_{2}}{\theta_{2}^{\prime }-\theta_{2}}, \end{array}$$

(32) $$\begin{array}{cc} & f\left(\rho ,\theta \right)=\left(1-w\right)f\left(\rho_{1},\theta \right)+wf\left(\rho_{2},\theta \right),\;w=\frac{\rho -\rho_{1}}{\rho_{2}-\rho_{1}}. \end{array}$$

Thus, the datum in position (ρ,θ) of the interpolated data can be calculated as shown below:

(33) $$\begin{array}{c} \begin{array}{cc} f(\rho ,\theta )= & \left(1-w\right)\left(1-h\right)f\left(\rho_{1},\theta_{1}\right)+\left(1-w\right)hf\left(\rho_{1},\theta_{1}^{\prime }\right)\\ & +w\left(1-l\right)f\left(\rho_{2},\theta_{2}\right)+wlf\left(\rho_{2},\theta_{2}^{\prime }\right). \end{array} \end{array}$$

#### 4.2.3. Quantum Realization of Biarcuate Interpolation Method

Because the number of samples of polar angles on each circumference is different, the interpolation process of adaptive sampled-data becomes quite complicated. Assume that a ρ×8ρ (ρ=0,1,…,M−1) quantum data |D〉 is scaled up to a $\rho^{\prime }\times 8\rho^{\prime }$ ($\rho =0,1,\ldots ,M^{\prime }-1$) quantum data $|D^{\prime }\rangle$ based on biarcuate interpolation. The scale ratio is *r*, i.e., $M^{\prime }=rM$, where r∈N and represented by *k* qubits. For the sake of simplicity, the interpolation process of adaptive sampled-data is divided into two steps according to the log-polar radius. The first step is interpolation based on the arcuate interpolation method. The second step is interpolation based on the biarcuate interpolation method using the data interpolated in the previous step. The specific process is discussed below.

Step 1: Circuit realization of arcuate interpolation method.

**Figure 21:**
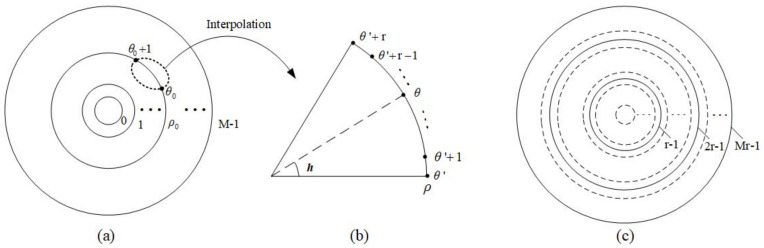
(**a**) The original data. (**b**) Schematic of arcuate interpolation. (**c**) The data after arcuate interpolation.

For each circumference with log-polar radius ρ in the scaled up data, if (ρ+1) is an integer multiple of *r*, i.e., $\rho +1=r(\rho_{0}+1)$, then the interpolated data is the corresponding circumference. As shown in Figure 21, $\rho_{0}$ and $\theta_{0}$ in (a) are the log-polar radius and polar angle corresponding to ρ and $\theta^{\prime }$ in (b), respectively. That is,

(34) $$\begin{array}{c} \rho_{0}\overset{enlarge}{\to }\rho ,\;\theta_{0}\overset{enlarge}{\to }\theta^{\prime },\;\theta_{0}+1\overset{enlarge}{\to }\theta^{\prime }+r. \end{array}$$

The data after arcuate interpolation is shown in Figure 21c as the solid line. In this step, the data on the dotted line has not been interpolated.

It can be seen from Figure 21b, there are (r−1) points between $\theta^{\prime }$ and $\left(\theta^{\prime }+r\right)$ that need to be interpolated. In order to get the data of these points, the value of $\rho_{0}$ and $\theta_{0}$ in the original data must be found first. Obviously,

(35) $$\begin{array}{c} r\left(\rho_{0}+1\right)=\rho +1,\;r\theta_{0}=\theta^{\prime },\;r\left(\theta_{0}+1\right)=\theta^{\prime }+r. \end{array}$$

Thus,

(36) $$\begin{array}{c} \rho_{0}=\frac{\rho +1}{r}-1=Q\left[\rho +1,r\right]\;-\;1,\;\theta_{0}=\frac{\theta^{\prime }}{r}=Q\left[\theta^{\prime },r\right]=Q\left[\theta ,r\right], \end{array}$$

where $\theta =\theta^{\prime },\theta^{\prime },\ldots ,\theta^{\prime }+r-1$ and Q[A,B] is the quotient of *A* divided by *B* in division with remainder. It is easy to get $h=\frac{R[\theta ,r]}{r}$, where R[A,B] is the remainder of *A* divided by *B* in division with remainder. Therefore, combining Equations (28) and (36), the value f(ρ,θ) in position (ρ,θ) of the interpolated data in Figure 21b can be calculated to arcuate interpolation according to the following equation:

(37) $$\begin{array}{ccc} f(\rho ,\theta ) & \;=\; & \left(1-h\right)f\left(\rho_{0},\theta_{0}\right)+hf\left(\rho_{0},\theta_{0}+1\right)\\ & \;=\; & \;\frac{r\;-\;R[\theta ,r]}{r}f\left(Q\left[\rho +1,r\right]\;-\;1,Q\left[\theta ,r\right]\right)+\frac{R[\theta ,r]}{r}f\;\left(Q\left[\rho \;+\;1,r\right]\;-\;1,Q\left[\theta ,r\right]\;+\;1\right)\\ & \;= & \;\frac{\left(\;r\;-\;R[\theta ,r])f\;(Q[\rho \;+\;1,r]\;-\;1,Q[\theta ,r])+R[\theta ,r]f\;(Q[\rho \;+\;1,r]\;-\;1,Q[\theta ,r]\;+\;1\right)}{r}, \end{array}$$

In order to prepare the data information f(ρ,θ) in position (ρ,θ) of the resulting data, the data information $f(\rho_{0},\theta_{0})$ and $f(\rho_{0},\theta_{0}+1)$ in positions $\left(\rho_{0},\theta_{0}\right)$ and $\left(\rho_{0},\theta_{0}+1\right)$ of the original data need to be prepared first. To achieve the data information for the corresponding position, two quantum operators $\Omega_{\rho_{0},\theta_{0}}$ and $\Omega_{\rho_{0},\theta_{0}+1}$ are used to achieve the original data information, respectively. The quantum operator $\Omega_{\rho ,\theta }$ can realize the aim of assigning the data information as follows:

(38) $$\Omega_{\rho ,\theta }{|0\rangle }^{⊗q}=\overset{q-1}{\underset{t=0}{⨂}}\left(\Omega_{\rho \theta }^{t}|0\rangle \right)=\overset{q-1}{\underset{t=0}{⨂}}|0⊕s_{\rho \theta }^{t}\rangle =\overset{q-1}{\underset{t=0}{⨂}}|s_{\rho \theta }^{t}\rangle =|f\left(\rho ,\theta \right)\rangle .$$

Due to the quantum superposition, all data are processed simultaneously, so all original data must be assigned. Let the assignment operation $\Omega =\sum_{\rho =0}^{M-1}\sum_{\theta =0}^{8\rho +7}\Omega_{\rho ,\theta }$, then the time complexity of the assignment operation is $O(qmM^{2})$.

**Figure 22:**
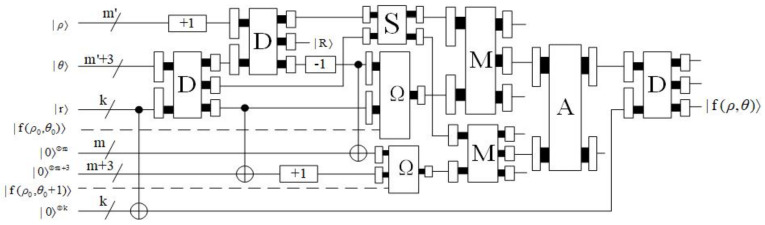
The quantum circuit of arcuate interpolation.

The quantum circuit of arcuate interpolation is shown in Figure 22, where $m=\left\{\begin{array}{cc} \left\lceil \log_{2}M\right\rceil , & M>1;\\ 1, & M=1, \end{array}\right.$ and $m^{\prime }=\left\{\begin{array}{cc} \left\lceil \log_{2}M^{\prime }\right\rceil , & M^{\prime }>1;\\ 1, & M^{\prime }=1, \end{array}\right.$. If the auxiliary bits are less than the input bits in multiple *C*-NOT operations, only the lowest bits of the input bits are copied. The dotted line serves no purpose but as an indicator. When R=R[ρ+1,r]=0, that is, when (ρ+1) is an integer multiple of *r*, the output f(ρ,θ) is the required data. Since the final output is the quotient without considering the remainder, f(ρ,θ) has an error of less than one. The time complexity of arcuate interpolation is $O({m^{\prime }}^{3}+qmM^{2})$.

Step 2: Circuit realization of biarcuate interpolation method.

**Figure 23:**
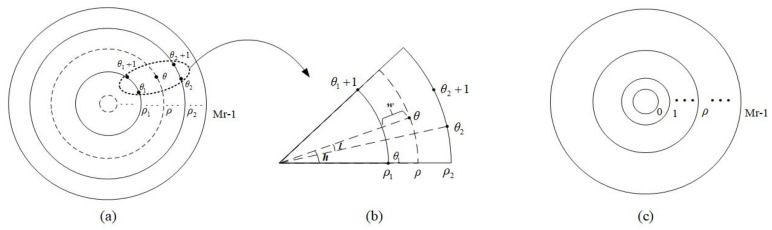
(**a**) The data after arcuate interpolation. (**b**) Schematic of biarcuate interpolation. (**c**) The scaled up data.

As shown in Figure 23, this step interpolates the part which is not interpolated in Step 1, that is, the data in the dotted part of Figure 21c or Figure 23a. Figure 23b shows the specific schematics of biarcuate interpolation for adaptive sampled-data, where points $\left(\rho_{1},\theta_{1}\right)$, $\left(\rho_{1},\theta_{1}+1\right)$, $\left(\rho_{2},\theta_{2}\right)$ and $\left(\rho_{2},\theta_{2}+1\right)$ in Figure 23a are the four closest points to (ρ,θ). Figure 23c describes the data after biarcuate interpolation, which is the scaled up data.

It is obvious that the four interpolated data $f(\rho_{1},\theta_{1})$, $f(\rho_{1},\theta_{1}+1)$, $f(\rho_{2},\theta_{2})$ and $f(\rho_{2},\theta_{2}+1)$ from the previous step are needed to complete the interpolation for this step. The mathematical expression is as follows:

(39) $$\begin{array}{c} \begin{array}{cc} f(\rho ,\theta )= & \left(1-w\right)\left(1-h\right)f\left(\rho_{1},\theta_{1}\right)+\left(1-w\right)hf\left(\rho_{1},\theta_{1}+1\right)\\ & +w\left(1-l\right)f\left(\rho_{2},\theta_{2}\right)+wlf\left(\rho_{2},\theta_{2}+1\right). \end{array} \end{array}$$

From Step 1, $\left(\rho_{1}+1\right)$ and $\left(\rho_{2}+1\right)$ are both integer multiples of *r*, assuming that:

(40) $$\begin{array}{c} \rho_{1}+1=kr,\;\rho_{2}+1=\left(k+1\right)r. \end{array}$$

where $k=Q[\rho_{1}+1,r]$. That is:

(41) $$\begin{array}{c} \rho_{1}=rk-1=rQ\left[\rho +1,r\right]-1, \end{array}$$

(42) $$\begin{array}{c} \rho_{2}=rk+r-1=rQ\left[\rho +1,r\right]+r-1, \end{array}$$

(43) $$\begin{array}{c} \rho_{2}-\rho_{1}=r. \end{array}$$

Oobviously,

(44) $$\begin{array}{c} w=\frac{R[\rho +1,r]}{\rho_{2}-\rho_{1}}=\frac{R[\rho +1,r]}{r}. \end{array}$$

Because there are 8(ρ+1) polar angles on each circumference with log-polar radius ρ, and the common multiple of $8(\rho_{1}+1)$ and 8(ρ+1) is $8\left(\rho +1\right)\left(\rho_{1}+1\right)$. The principle of common multiples is used, there are:

(45) $$\begin{array}{c} \theta_{1}=Q\left[\theta \left(\rho_{1}+1\right),\left(\rho +1\right)\right]=Q\left[\theta rQ\left[\rho +1,r\right],\rho +1\right], \end{array}$$

(46) $$\begin{array}{c} h=\frac{R[\theta \left(\rho_{1}+1\right),\left(\rho +1\right)]}{\rho +1}=\frac{R[\theta rQ[\rho +1,r],\rho +1]}{\rho +1}. \end{array}$$

In the same manner,

(47) $$\begin{array}{c} \theta_{2}=Q\left[\theta \left(\rho_{2}+1\right),\left(\rho +1\right)\right]=Q\left[\theta \left(rQ\left[\rho +1,r\right]+r\right),\rho +1\right], \end{array}$$

(48) $$\begin{array}{c} l=\frac{R[\theta \left(\rho_{2}+1\right),\left(\rho +1\right)]}{\rho +1}=\frac{R[\theta (rQ[\rho +1,r]+r),\rho +1]}{\rho +1}. \end{array}$$

Thus, the Equation (39) can be rewritten as:

(49) $$\begin{array}{c} \begin{array}{cc} f(\rho ,\theta )= & [\left(r-R[\rho +1,r]\right)\left(\rho +1-R[\theta rQ[\rho +1,r],\rho +1]\right).\\ & \left.\times f(rQ[\rho +1,r]-1,Q[\theta rQ[\rho +1,r],\rho +1])\right.\\ & \left.+\left(r-R[\rho +1,r]\right)R\left[\theta rQ\left[\rho +1,r\right],\rho +1\right]\right.\\ & \left.\times f(rQ[\rho +1,r]-1,Q[\theta rQ[\rho +1,r],\rho +1]+1)\right.\\ & \left.+R\left[\rho +1,r\right]\left(\rho +1-R[\theta (rQ[\rho +1,r]+r),\rho +1]\right)\right.\\ & \left.\times f(rQ[\rho +1,r]+r-1,Q[\theta (rQ[\rho +1,r]+r),\rho +1])\right.\\ & \left.+R[\rho +1,r]R[\theta (rQ[\rho +1,r]+r),\rho +1]\right.\\ & \times f(rQ[\rho +1,r]+r-1,Q[\theta (rQ[\rho +1,r]+r),\rho +1]+1)]÷r÷(\rho +1). \end{array} \end{array}$$

**Figure 24:**
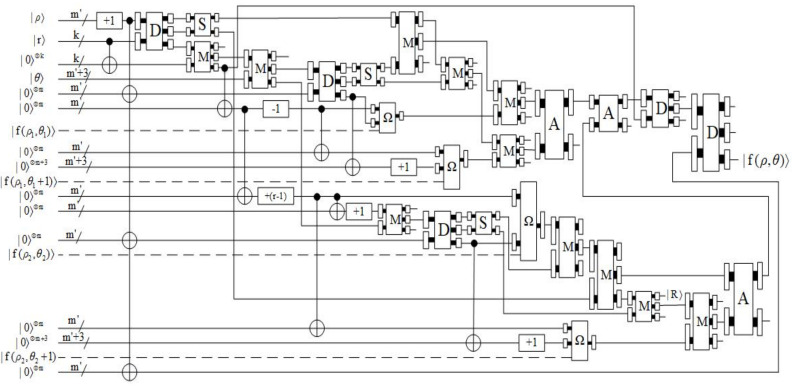
The quantum circuit of biarcuate interpolation.

As shown in Figure 24, the quantum circuit of biarcuate interpolation is given, where: $m^{\prime }=\left\{\begin{array}{cc} \left\lceil \log_{2}M^{\prime }\right\rceil , & M^{\prime }>1;\\ 1, & M^{\prime }=1, \end{array}\right.$ and the +(r−1) operation can be realized by (r−1) plus one operation. The dotted line serves no purpose but as an indicator. R=R[ρ+1,r] represents the remainder of (ρ+1) divided by *r*. When R≠0, the output f(ρ,θ) is the required data. Similarly, f(ρ,θ) has an error of less than one. However, due to the characteristics of the adaptive sampling method, there may be additional errors, which will be explained in detail in the next subsection. The time complexity of biarcuate interpolation is $O({m^{\prime }}^{3}+rqm^{\prime }M^{2})$. Therefore, the time complexity of the whole interpolation process is $O({m^{\prime }}^{3}+rqm^{\prime }M^{2})$.

In classical computers, the algorithm complexity is mainly evaluated by two parameters, one is the number of rows of pre-cached data required to complete interpolation, and the other is the number of operation steps such as addition, subtraction, multiplication, division and displacement required to complete interpolation. Here, the time complexity of the proposed algorithm is estimated by 1-bit addition and subtraction as the unit of operation on classical computers. The m×n multiplication consists of O(mn) additions, and the n×n division can be decomposed into $O(n^{2})$ subtractions, assuming that all of the original data is known. By Equations (37) and (49), the time complexity of arcuate interpolation and biarcuate interpolation are $O(\left({m^{\prime }}^{2}+k^{2}q^{2}\right)rM^{2})$ and $O(\left(k^{2}+q^{2}\right)rq^{2}{m^{\prime }}^{2}M^{2})$, respectively. Therefore, the time complexity of the whole interpolation process in classical computers is $O(\left(k^{2}+q^{2}\right)rq^{2}{m^{\prime }}^{2}M^{2})$. Obviously, the time complexity is much higher than that of quantum computers.

### 4.3. Example Verification

In this section, the sample data in Figure 4 is used to demonstrate the specific scaling-up process. For the ρ×8ρ (ρ=0,1) data, we scale it up three times to become a $\rho^{\prime }\times 8\rho^{\prime }$ ($\rho^{\prime }=0,1,\ldots ,5$) dataset. When $\rho^{\prime }=2$ or 5, $\left(\rho^{\prime }+1\right)$ is an integer multiple of three. Therefore, the data when $\rho^{\prime }=2$ and 5 are interpolated first. When $\rho^{\prime }=2$, the Equation (37) can be rewritten as:

(50) $$\begin{array}{c} f\left(2,\theta \right)=\frac{\left(3-R[\theta ,3]\right)f\left(0,Q\left[\theta ,3\right]\right)+R\left[\theta ,3\right]f\left(0,Q\left[\theta ,3\right]+1\right)}{3}, \end{array}$$

where θ=0,1,…,23. The specific operation is as follows:

(51) $$\begin{array}{c} f(2,0)=f(0,0)=0, \end{array}$$

(52) $$\begin{array}{c} f\left(2,1\right)=\frac{2f(0,0)+f(0,1)}{3}=10,\\ \vdots \end{array}$$

(53) $$\begin{array}{c} f\left(2,23\right)=\frac{f(0,7)+2f(0,8)}{3}=\frac{f(0,7)+2f(0,0)}{3}=64. \end{array}$$

**Figure 25:**
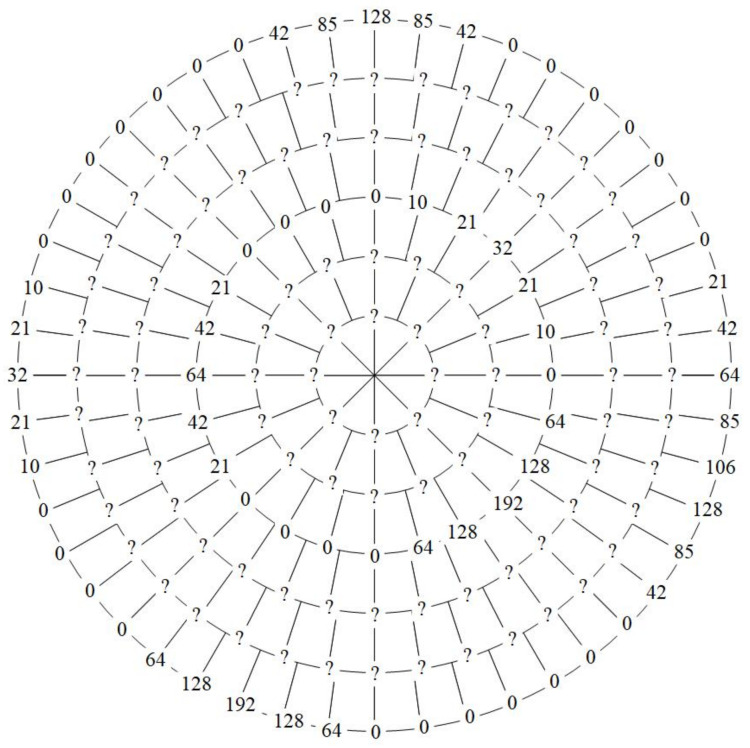
The data after arcuate interpolation.

Owing to the characteristics of the plus one module, f(0,8)=f(0,0). In the same manner, the data when $\rho^{\prime }=5$ can be achieved. The data after arcuate interpolation are shown in Figure 25.

**Figure 26:**
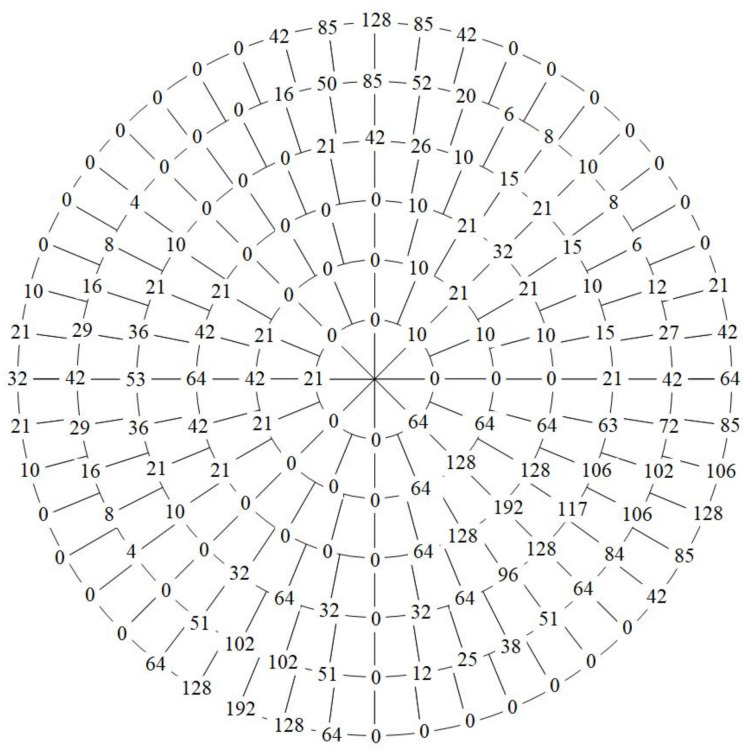
The sample data in Figure 4 is magnified three times.

Then, the rest of the data can be calculated from the data already obtained by Equation (50). When $\rho^{\prime }>r=3$, the data that need to be interpolated can be easily calculated. For example, when $\rho^{\prime }=4$, the Equation (49) can be rewritten as:

(54) $$\begin{array}{ccc} f(4,\theta )\; & = & \;[\left(5-R[3\theta ,5]\right)f\left(2,Q\left[3\theta ,5\right]\right)+R\left[3\theta ,5\right]f\left(2,Q\left[3\theta ,5\right]+1\right). \end{array}$$

(55) $$\begin{array}{ccc} & & \;+2\left(5-R[6\theta ,5]\right)f\left(5,Q\left[6\theta ,5\right]\right)+2R\left[6\theta ,5\right]f\left(5,Q\left[6\theta ,5\right]+1\right)]÷15, \end{array}$$

where θ=0,1,…,39. And the specific operation is as follows:

(56) $$\begin{array}{c} f\left(4,0\right)=\frac{5f(2,0)+10f(5,0)}{15}=42, \end{array}$$

(57) $$\begin{array}{c} f\left(4,1\right)=\frac{2f(2,0)+3f(2,1)+8f(5,1)+2f(5,2)}{15}=27,\\ \vdots \end{array}$$

(58) $$\begin{array}{c} f\left(4,39\right)=\frac{3f(2,23)+2f(2,24)+2f(5,46)+8f(5,47)}{15}=72, \end{array}$$

where f(2,24)=f(2,0)=0. However, when $\rho^{\prime }<r=3$, the process becomes a little more complicated. When $\rho^{\prime }=0$, the Equation (49) turns into:

(59) $$f\left(0,\theta \right)=\frac{2f(-1,0)+f(2,3\theta )}{3}=\frac{f(2,3\theta )}{3},\;\theta =0,1,\ldots ,7,$$

where f(−1,0) should be the data of the pole in the log-polar coordinates. However, the adaptive sampling method does not sample at the poles; thus, the characteristics of the minus one module are f(−1,0)=f(7,0). In this example, f(7,0) is a redundant datum that has not been prepared, so f(7,0)=0. If f(7,0)≠0, there is a certain error. The effect of all the data after biarcuate interpolation is shown in Figure 26.

## 5. Conclusions

In the existing quantum data representation models, the two variables representing positions are independent of each other. However, in the quantum data representation model proposed in this paper, the upper limit of the sampling number of the polar angles is related to the log-polar radius. This is an inspiration for the future research work about the representation of irregularly shaped data.

The quantum scaling up algorithm with integer scaling ratio based on biarcuate interpolation and its circuit of adaptive sampled-data in log-polar coordinates are also given in this paper. In the quantum scaling up circuit, the operation modules required are constructed on the basis of the existing methods in the references. Meanwhile, the feasibility of the quantum algorithm is also verified by an example. However, the quantum scaling up algorithm still has certain shortcomings, and the quantum scaling down circuit has not been given. That’s one of the most important things we’ll be looking at in the future. In addition, how to change the scaling ratio from integer multiples to real multiples and how to optimize the circuit are also problems worth studying in future work.

## References

[1] Feynman R.P. (1982). Simulating physics with computers. Int. J. Theor. Phys.

[2] Shor P.W. Algorithms for quantum computation: Discrete logarithms and factoring. Proceedings of the 35th Annual Symposium on Foundations of Computer Science.

[3] Grover L.K. A fast quantum mechanical algorithm for database search. Proceedings of the 28th Annual ACM Symposium on the Theory of Computing (STOC).

[4] Venegas-Andraca S.E., Bose S. Storing, processing and retrieving an image using quantum mechanics. Proceedings of the SPIE 5105, Quantum Information and Computation.

[5] Latorre J.I. (2005). Image compression and entanglement. arXiv.

[6] Venegas-Andraca S.E., Ball J.L. (2010). Processing images in entangled quantum systems. Quantum Inf. Process..

[7] Le P.Q., Dong F.Y., Hirota K. (2011). A flexible representation of quantum images for polynomial preparation, image compression, and processing operations. Quantum Inf. Process..

[8] Zhang Y., Lu K., Gao Y.H., Wang M. (2013). NEQR: A novel enhanced quantum representation of digital images. Quantum Inf. Process..

[9] Zhang Y., Lu K., Gao Y.H., Xu K. (2013). A novel quantum representation for log-polar images. Quantum Inf. Process..

[10] Zhang R., Xu M.Y., Lu D.Y. (2020). A generalized floating-point quantum representation of 2-D data and their applications. Quantum Inf. Process..

[11] Wang B., Hao M.Q., Li P.C., Liu Z.B. (2020). Quantum representation of indexed images and its applications. Int. J. Theor. Phys..

[12] Le P.Q., Iliyasu A.M., Dong F., Hirota K. (2010). Fast Geometric Transformations on Quantum Images. IAENG Int. J. Appl. Math..

[13] Jiang N., Wu W., Wang L., Zhao N. (2015). Quantum image pseudocolor coding based on the density-stratified method. Quantum Inf. Process..

[14] Jiang N., Ji Z., Li H., Wang J. (2021). Quantum image interest point extraction. Mod. Phys. Lett. A.

[15] Jiang N., Wang J., Mu Y. (2015). Quantum image scaling up based on nearest-neighbor interpolation with integer scaling ratio. Quantum Inf. Process..

[16] Zhou R.G., Hu W.W., Fan P., Lan H. (2017). Quantum realization of the bilinear interpolation method for NEQR. Sci. Rep-UK.

[17] Li P.C., Liu X. (2018). Bilinear interpolation method for quantum images based on quantum Fourier transform. Int. J. Quantum Inf..

[18] Zhou R.G., Cheng Y.D., Lan H., Liu X.A. (2019). Quantum watermarking algorithm based on chaotic affine scrambling. Int. J. Quantum Inf..

[19] Li P.C., Xiao H., Li B.X. (2016). Quantum representation and watermark strategy for color images based on the controlled rotation of qubits. Quantum Inf. Process..

[20] Yan F., Abdullah M.I., Phuc Q.L., Sun B., Dong F.Y., Kaoru H. (2013). A parallel comparison of multiple pairs of images on quantum computers. Int. J. Innov. Comput. Appl. (IJICA).

[21] Matungka R. (2009). Studies on Log-Polar Transform for Image Registration and Improvements Using Adaptive Sampling and Logarithmic Spiral. Ph.D. Thesis.

[22] Wang D., Liu Z., Zhu Y., Li S. (2012). Design of Quantum Comparator Based on Extended General Toffoli Gates with Multiple Targets. Comput. Sci..

[23] Nielsen M.A., Chuang I.L. (2000). Quantum Computation and Quantum Information.

[24] Barenco A., Bennett C.H., Cleve R., Divincenzo D.P., Margolus N., Shor P., Sleator T., Smolin J., Weinfurter H. (1995). Elementary gates for quantum computation. Phys. Rev. A.

[25] Vedral V., Barenco A., Ekert A. (1996). Quantum networks for elementary arithmetic operations. Phys. Rev. A.

[26] Li P.C., Shi T., Zhao Y., Lu A. (2020). Design of threshold segmentation method for quantum image. Int. J. Theor. Phys..

[27] Khosropour A., Aghababa H., Forouzandeh B. Quantum Division Circuit Based on Restoring Division Algorithm. Proceedings of the 2011 Eighth International Conference on Information Technology.

